# Supplementary material for: Signature-driven repurposing of Midostaurin for combination with MEK1/2 and KRASG12C inhibitors in lung cancer
Source: Nat Commun. 2023 Oct 10;14:6332. doi: 10.1038/s41467-023-41828-z (PMC10564741; doi:10.1038/s41467-023-41828-z)

SUPPLEMENTARY INFORMATION

‘‘Signature-driven repurposing of Midostaurin for combination with MEK1/2 and KRASG12C inhibitors in lung cancer’’ (Macaya I. and Roman M. et al.).

Supplementary tables

Suppl. Table 1. List of repurposed drugs for pairwise screening. List of repurposed drugs, intended targets and final selected drugs for the pairwise screen in mutant *KRAS* lung cancer cell lines.

PREDICTED DRUG NAME	FUNCTION	COMPOUND USED	REPURPOSING SCORE
**Dabrafenib**	BRAF, BRAFV600E and CRAF inhibitor	Dabrafenib	-0.4048
**U0126**	MEK1/2 inhibitor	Trametinib	-0.4036
**AZ-628**	Wild-type CRAF and BRAFV600E inhibitor	Dabrafenib	-0.3978
**AS-703026 (Pimasertib)**	MEK1/2 inhibitor	Trametinib	-0.3871
**PD-198306**	MEK1/2 inhibitor	Trametinib	-0.3857
**SA-25547**	Info not available		-0.3316
**BRD-K10899576**	MEK5 kinase 2 inhibitor	BIX02189	-0.3286
**BRD-K10846167**	WEE1, NR2F2 (NFkB), CK1d and FLT3 inhibitor	Adavosertib	-0.3179
**Neratinib**	HER2 and EGFR inhibitor	Neratinib	-0.3120
**Lestaurtinib**	JAK, FLT3 inhibitor	Lestaurtinib	-0.3090
**Panobinostat**	HDAC inhibitor	Panobinostat	-0.3007

Supplementary figures

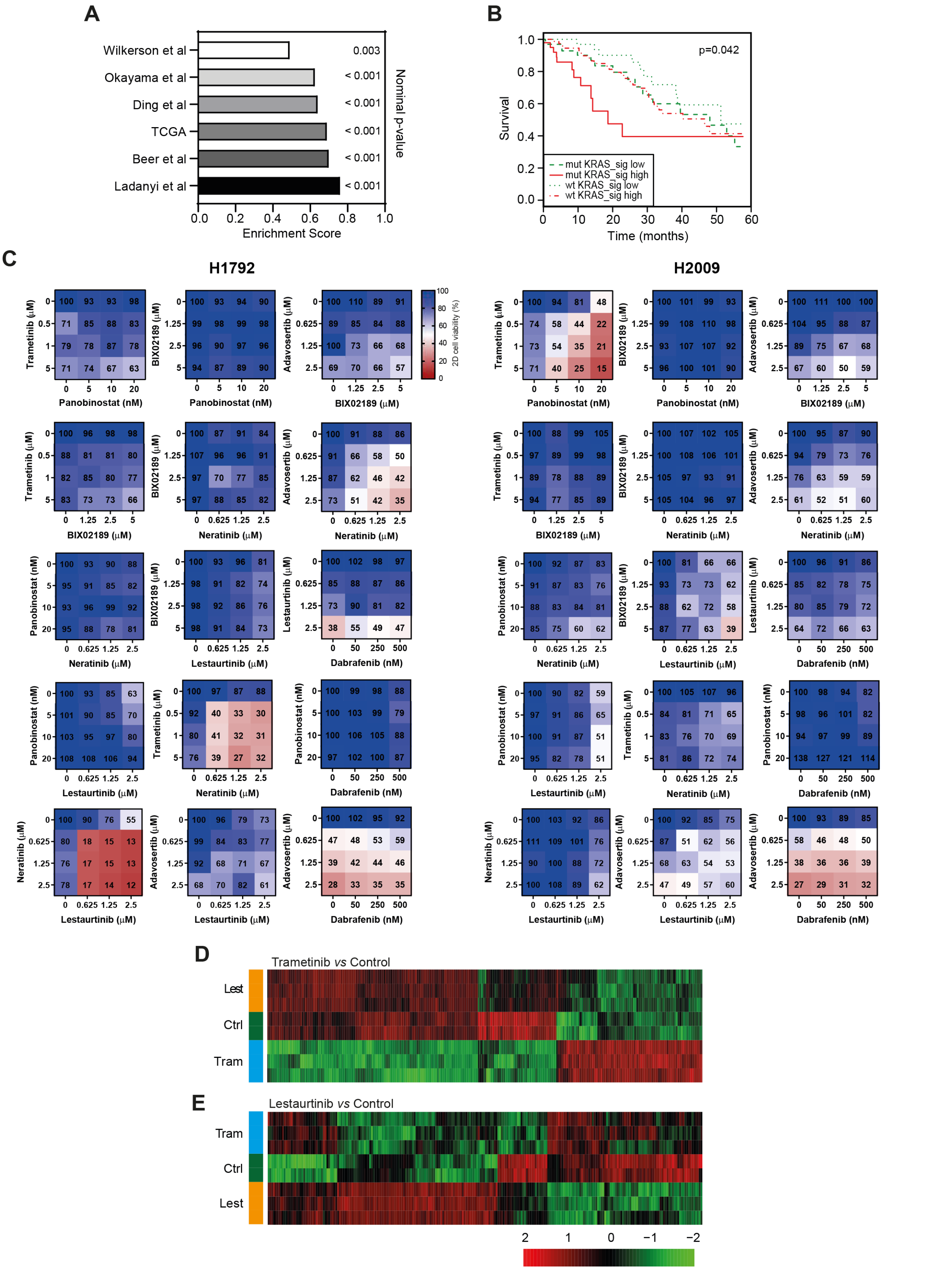

Suppl. Fig. 1. Synergistic drug combinations for mutant *KRAS* lung cancer obtained through a drug repurposing-based strategy. A. Enrichment score of the upregulated genes of the

iKRASsig in mut *KRAS* lung cancer patients of several lung cancer data sets (1-6). B. Kaplan-Meier plot showing overall survival of lung cancer patients from TCGA database as a function of iKRASsig expression and *KRAS* mutational status. C. Cell viability percentage of H1792 and H2009 cells treated with different concentrations of Trametinib, BIX02189, Neratinib, Lestaurtinib, Dabrafenib, Adavosertib and Panobinostat, individually or in a pairwise manner. D-E. Heatmap of genes differently expressed upon treatment of H1792 cells with Trametinib (Tram; D), or with Lestaurtinib (Lest; E) (logFC ±1, B > 0). Ctrl: control.

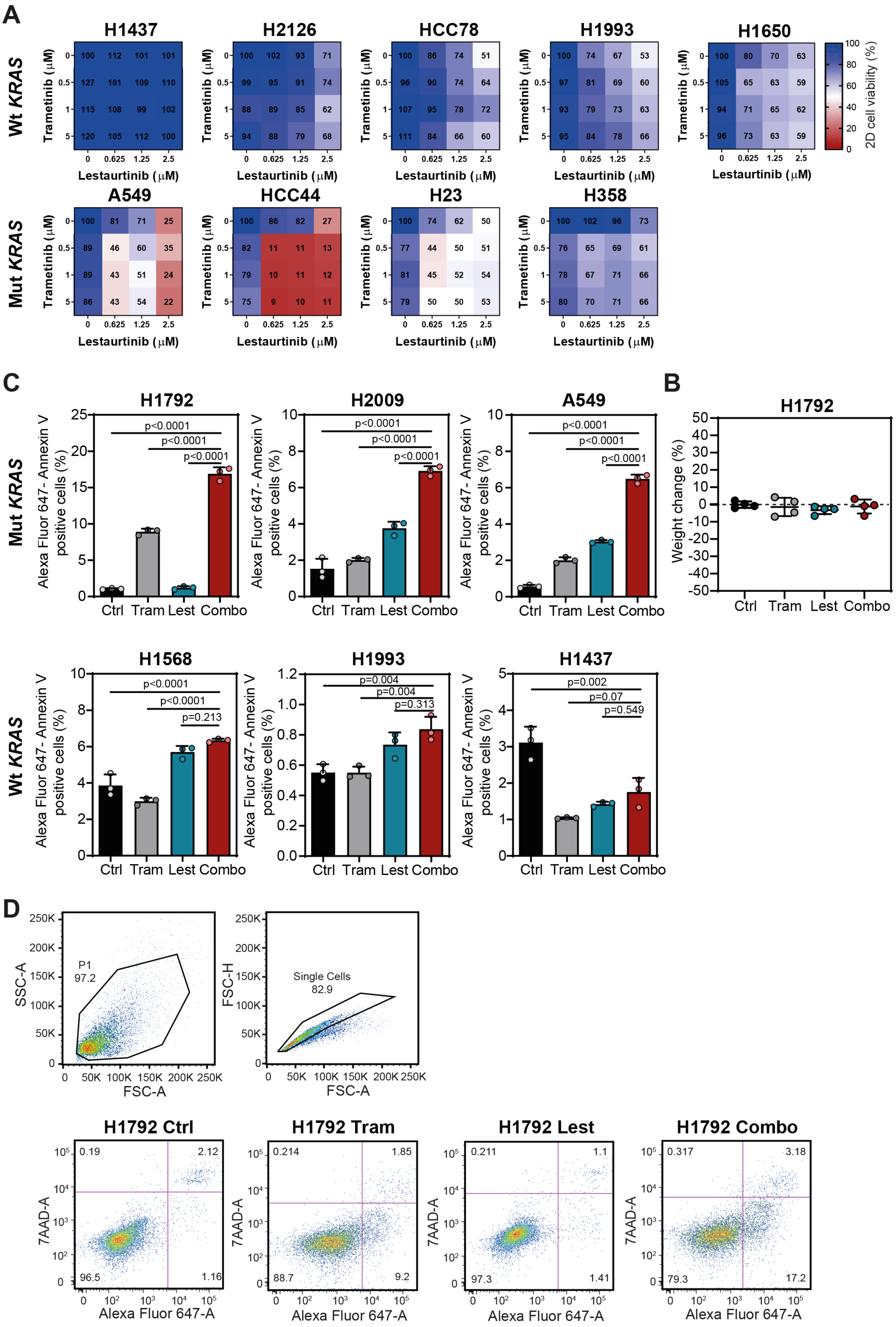

Suppl. Fig. 2. Trametinib and Lestaurtinib combination is preferentially effective in mutant *KRAS* lung cancer cells compared to wild type *KRAS* ones. A. Heatmaps showing cell

viability percentage of wild type (wt) *KRAS* (H1437, H2126, HCC78, H1993 and H1650) and mutant (mut) *KRAS* (A549, HCC44, H23 and H358) cells treated with different concentrations of Trametinib (MEKi) and Lestaurtinib (FLT3i), individually or in combination, as indicated. B. Percent weight change of mice administered single and double treatments the last day of experiment compared to the first day of treatment (data: mean +/- SD). C. Annexin V positive cells' percentage in mut *KRAS* (H1792, H2009 and A549) and wt *KRAS* (H1568, H1993 and H1437) cell lines after 24 h of drug treatment. Ctrl: untreated cells; Tram: 0.5 μM; Lest: 0.625 μM; Combo: dual treatment (n: 3 independent experiments; data: mean +/- SD; test: oneway ANOVA, Tukey's adjustment). D. Gating strategy for cytometry data. Up: FSC / SSC plots to select alive and single cells are shown. Down: Representative apoptosis analysis (7AAD *vs* Alexa Fluor 647-Annexin V) in H1792 cell line treated with indicated drugs is shown.

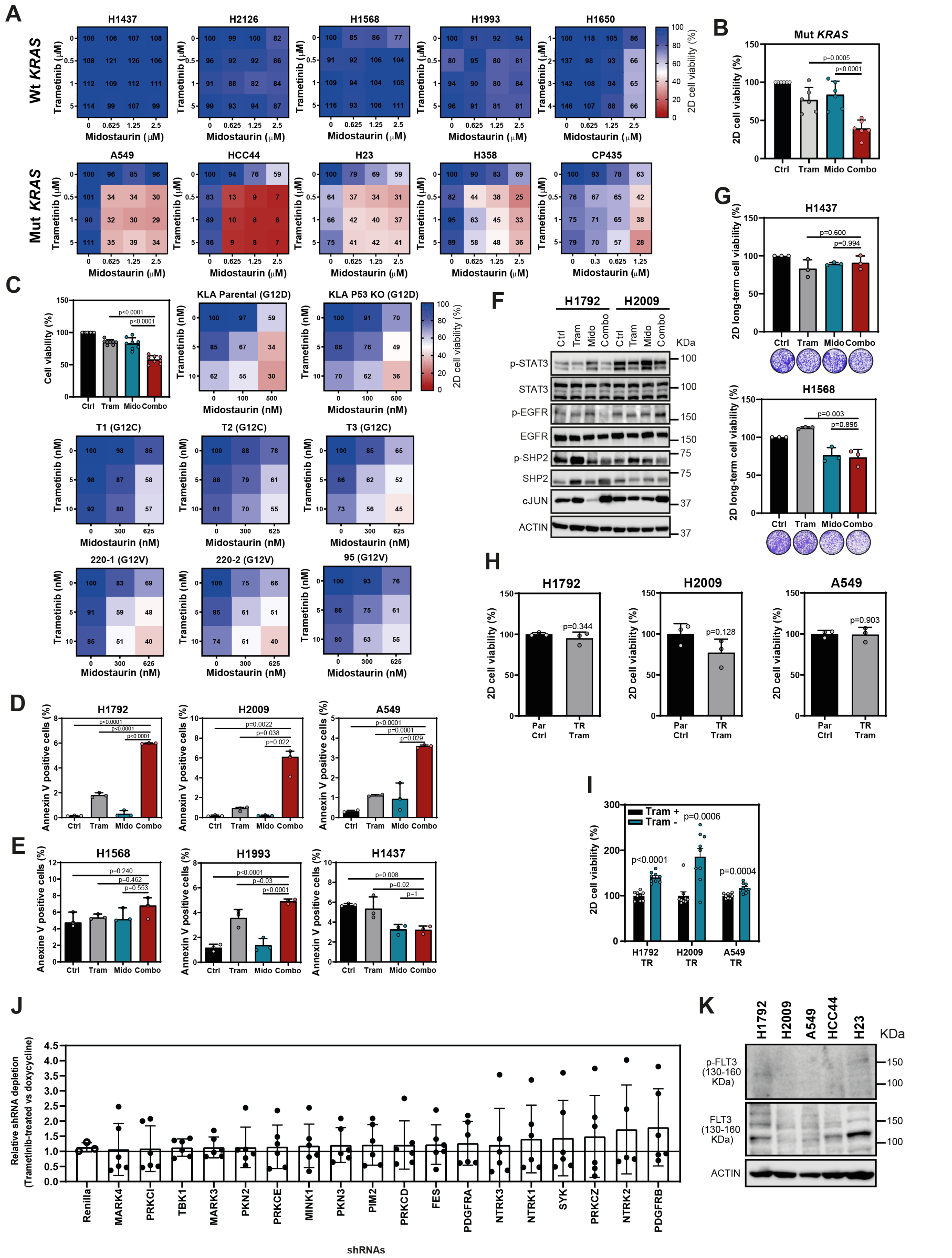

Suppl. Fig. 3. Consequences of Trametinib and Midostaurin dual treatment on wild type and mutant *KRAS* lung cancer cell lines. A. Heatmaps showing percentage of cell viability of

wild type (wt) *KRAS* (H1437, H2126, H1568, H1993 and H1650) and mutant (mut) *KRAS* (A549, HCC44, H23, H358 and CP435) cells treated with different concentrations of Trametinib (Tram: MEKi) and Midostaurin (Mido: FLT3i), individually or in combination, as indicated. B. Effects of Tram and Mido combination on cell viability of mut *KRAS* (H1792, H2009, A549, HCC44, H23, H358) cells (5-day treatment). Tram: 5 nM; Mido: 100 nM (data: mean +/- SD; test: one-way ANOVA, Tukey's adjustment). C. Effect of Tram and Mido combination on cell viability of mouse lung cancer cell lines with different *KRas* mutations: *G12D*: KLA Parental, KLA *p53ko*; *G12C*: T1, T2, T3; *G12V*: 220-1, 220-2, 95 (72-h drug treatment). Heatmaps are average of 3 experiments (n: 8 cell lines/biological replicates; data: mean +/- SD; test: one-way ANOVA, Tukey's adjustment). D and E. Annexin V positive cells' percentage in mut *KRAS* (H1792, H2009 and A549; D) and wt *KRAS* (H1568, H1993 and H1437; E) cell lines 24 h after drug treatment Ctrl: untreated cells; Tram: 0.5 μM; Mido: 0.625 μM (n: 3 independent experiments; data: mean +/- SD; test: one-way ANOVA, Tukey's adjustment). F. Western blotting of indicated proteins in H1792 and H2009 cell lines. Ctrl, control; Tram, Trametinib; Mido, Midostaurin; Combo, combined therapy (loading control: ACTIN). G. Long-term effects of Tram and Mido combination on cell viability of wt *KRAS* H1437 and H1568 lung cancer cells after 10-day treatment (n: 3 independent experiments; data: mean +/- SD; test: one-way ANOVA, Tukey's adjustment). Representative images of crystal violet-stained control (DMSO-treated), Tram-, Mido- and combo-treated cells. H. Relative percentage of untreated parental (Par control) and Tram-resistant (TR; undergoing continuous Tram treatment) cells from H1792, H2009 and A549 cell lines (data: mean +/- SD; test: t-test). I. Percent cell viability of H1792-TR, H2009-TR and A549-TR cells in the presence or absence of Tram (72-h treatment; n: 3 independent experiments; data: mean +/- SD; test: t-test). J. Relative depletion of shRNAs targeting mtPKCi Midostaurin putative targets in Trametinib-treated

versus Doxycycline-treated H23 cells (data: mean +/- SD). K. Western blotting of pFLT3 and FLT3 proteins in mut *KRAS* LUAD cell lines (loading control: ACTIN).

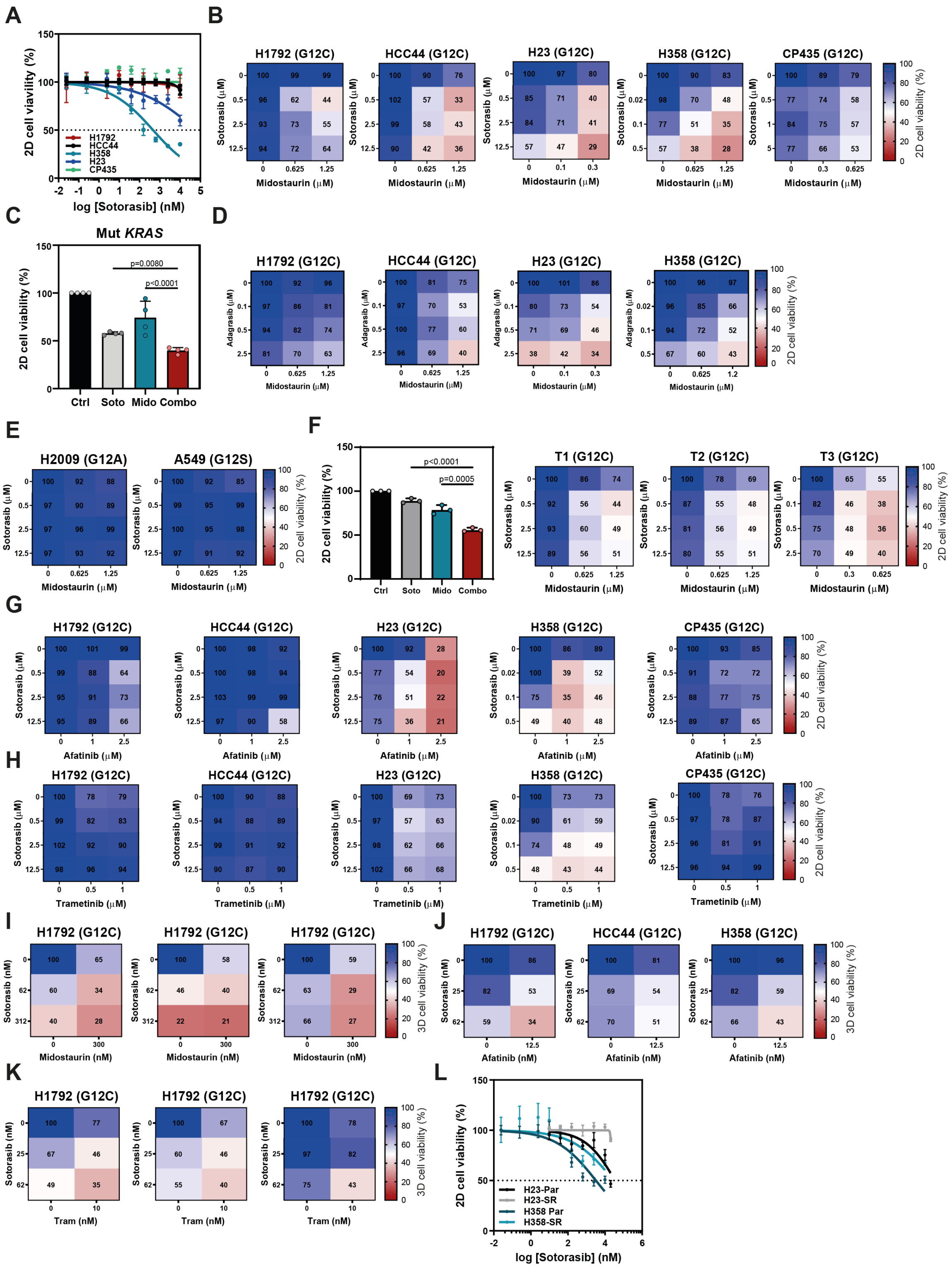

Suppl. Fig. 4. Sotorasib and Midostaurin combination has a synergistic effect on *KRASG12C* lung cancer cells. A. Percentage cell viability of mutant *KRASG12C* lung

cancer cells (H1792, HCC44, H23, H358 and CP435) after 3 days of exposure to increasing concentrations of Sotorasib (Soto; KRASi). Data: mean +/- SD. B. Percent cell viability of *KRASG12C* cell lines (H1792, HCC44, H23, H358 and CP435) treated with different concentrations of Sotorasib and Midostaurin (mtPKCi) individually or in combination. C. Effects of Sotorasib and Midostaurin combination on cell viability of mut *KRAS* (H1792, HCC44, H23 and H358) cells 5 days after drug treatment. Soto: 20-100 nM; Mido: 50-100 nM (data: mean +/- SD; test: t-test). D. Percent cell viability of *KRASG12C* cell lines (H1792, HCC44, H23 and H358) treated with different concentrations of Adagrasib (KRASi) and Midostaurin individually or in combination. E. Percent cell viability of H2009 (*KRASG12A*) and A549 (*KRASG12S*) cell lines treated with different concentrations of Sotorasib and Midostaurin. F. Left. Percent of cell viability of $Kras^{FSFG12C}$; $Trp53^{FRT//FRT}$ T1, T2 and T3 mouse cell lines 72 h after exposure to indicated treatments (data: mean +/- SD; test: one-way ANOVA, Tukey's adjustment). Right. Individual heatmap of percent cell viability of T1, T2 and T3 mouse cell lines treated with different concentrations of Sotorasib (Soto) and Midostaurin (Mido), individually or in combination. G-H. Percent cell viability of *KRASG12C* cell lines (H1792, HCC44, H23, H358 and CP435) treated with different concentrations of Sotorasib and Afatinib (EGFRi) (G) or Sotorasib and Trametinib (MEKi) (H), individually or in combination. I-K. Percent cell viability of *KRASG12C* cell lines (H1792, HCC44, and H358) grown in 3D conditions and treated with different concentrations of Sotorasib and Midostaurin (I), Sotorasib and Afatinib (EGFRi) (J) or Sotorasib and Trametinib (MEKi) (K), individually or in combination. L. Percentage cell viability of H23-Par, H358-Par (Par: parental), H23-SR and H358-SR (Sotorasib resistant) cell lines after 3 days of exposure to increasing concentrations of the KRASi. All heatmaps are average of 3 experiments (data: mean +/- SD).

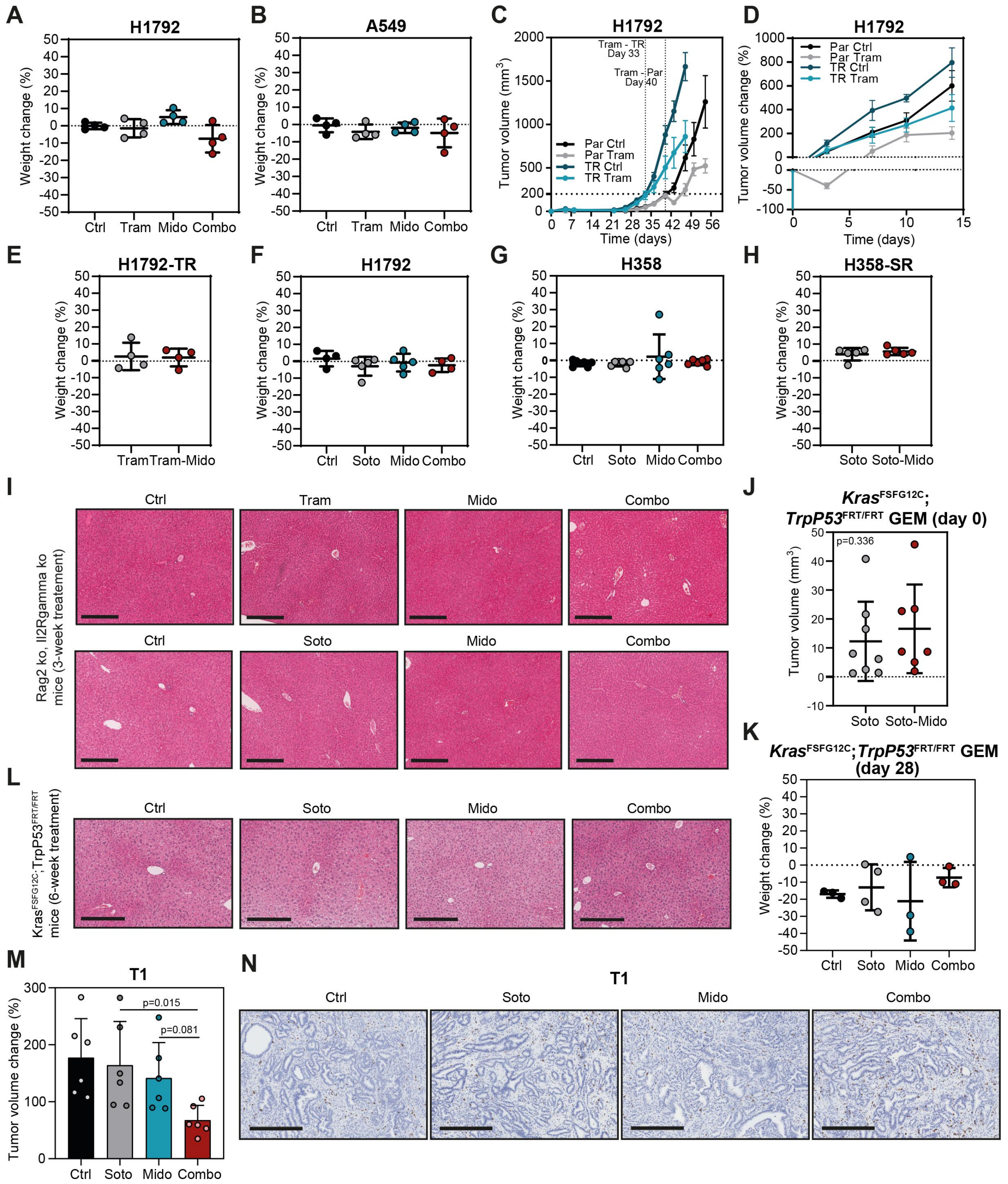

Suppl. Fig. 5. Midostaurin-based drug combinations show antitumor effects on treatment naïve and resistant mut *KRAS* lung tumors. A and B. Percent mouse weight change of subcutaneous growth experiments of H1792 (A) and A549 (B) cell lines. N: 4 Rag2$^{-/-}$; Il2γr$^{-/-}$ mice per group (data: mean +/- SD). C and D. Absolute growth (C) or percent fold change

growth (D) of parental (Par) H1792 and H1792-TR (Tram-resistant) cells over time when exposed to indicated treatments. Tram treatment started at day 33 for H1792-TR-derived xenografts and at day 40 for parental H1792-derived xenografts. Trametinib concentration: 1 mg/kg. N: 6 tumors per group in Rag2$^{-/-}$; Il2γr$^{-/-}$ mice (data: mean +/- SD). E. Percent weight change of in Rag2$^{-/-}$; Il2γr$^{-/-}$ mice injected with H1792-TR cells and exposed to indicated treatments. N: 4 Rag2$^{-/-}$; Il2γr$^{-/-}$ mice per group (data: mean +/- SD). F-H. Percent weight change of mice injected with H1792 (Parental; F), H358 (Parental; G), or H358-SR (Soto-resistant; H) cells and exposed to indicated treatments. N: 4-6 Rag2$^{-/-}$; Il2γr$^{-/-}$ mice per group (data: mean +/- SD). I. H&E-stained liver sections of Rag2$^{-/-}$; Il2γr$^{-/-}$ mice treated with indicated drugs for 3 weeks. Scale bar: 300 μm. J. Tumor volume from the *Kras*^{FSFG12C}*;* *Trp53*$^{FRT/FRT}$ driven LUAD model mice at the day of treatment start (data: mean +/- SD; test: Mann-Whitney). K. Percent weight change of *Kras*^{FSFG12C}*; Trp53*$^{FRT/FRT}$ mice exposed to indicated treatments (data: mean +/- SD). L. H&E-stained liver sections of *Kras*$^{\mathrm{FSFG12C}}$; *TrpP53*$^{\mathrm{FRT/FRT}}$ mice treated with indicated drugs for 6 weeks. Scale bar: 300 μm. M. Percent fold change volume of T1-derived xenografts in F1 C57BL/6 x 129S4/Sv mice at the last day of the experiment after 7-day treatment with indicated drugs (Soto: 30 mg/kg; Mido: 25 mg/kg). N= 6 tumors per group (test: one-way ANOVA, Dunnett's adjustment). N. CD8 stained tumor sections from tumors in (N). Scale bar: 300 μm.

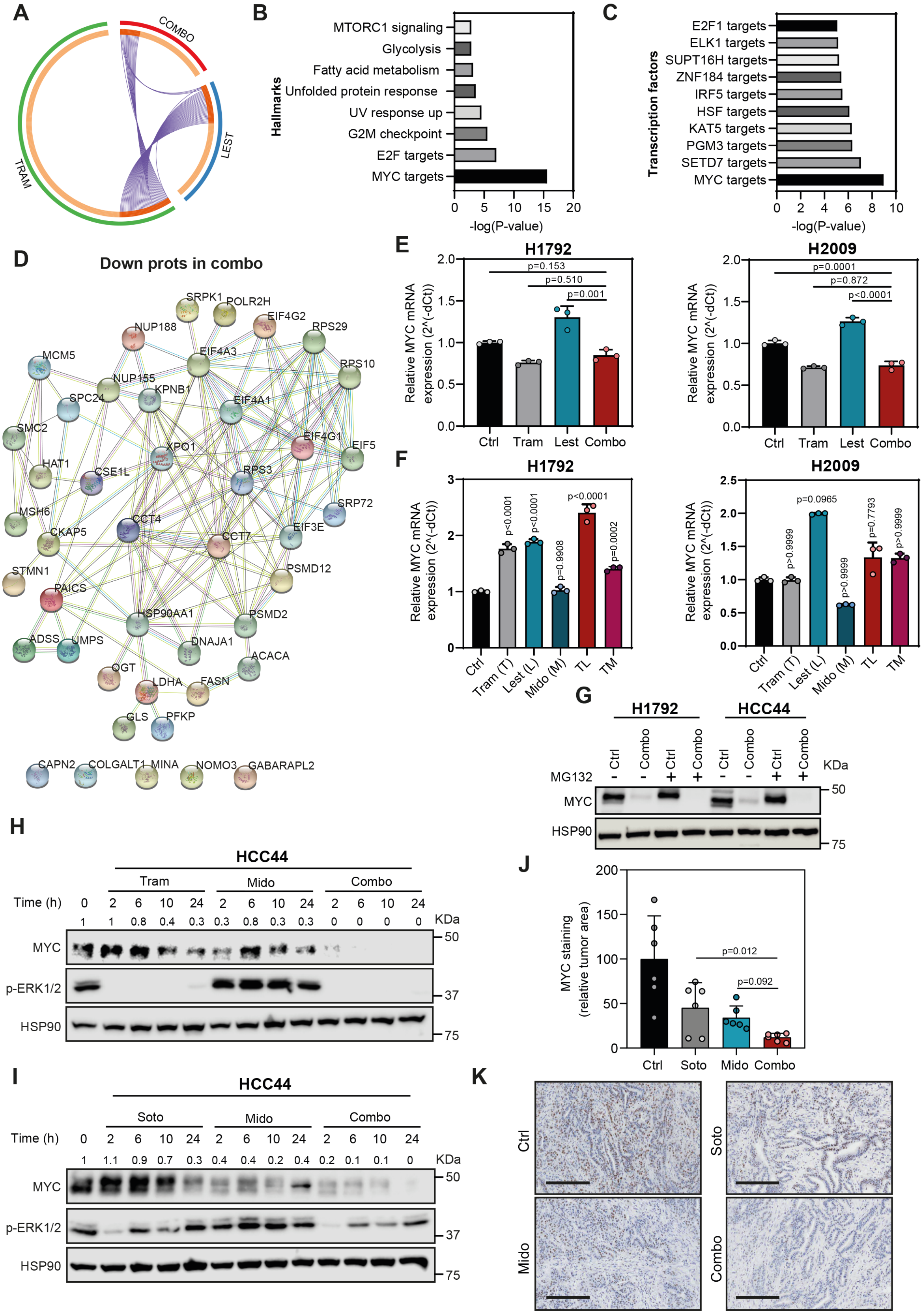

Suppl. Fig. 6. MtPKCi-based drug combinations result in MYC protein decrease. A. Circos plot of dysregulated proteins obtained in H1792 cell line 48 h after exposure to Trametinib

(Tram), Lestaurtinib (Lest) or both. B-C. Enrichment of downregulated proteins from the Tram plus Lest condition in the Hallmarks (B) and Transcription Factors (C) features of the Molecular Signature Data Base (MSigDB). D. Protein-protein interaction network of proteins significantly downregulated upon combined Tram and Lest administration in H1792 cells obtained by STRING. E and F. Relative MYC mRNA expression of H1792 and H2009 cell lines 24 h (E) or 48 h (F) after exposure to indicated treatments. Housekeeping for qPCR: GAPDH. Data: mean +/- SD. Test: one-way ANOVA, Tukey's adjustement (E); one-way ANOVA, Dunnet's adjustment (F, H1792); Kruskal-Wallis, Dunn'G. MYC protein expression of H1792 and HCC44 cell lines after treatment with Trametinib and Midostaurin combination for 48 h, and subsequently 6 h with or without proteasome inhibitor (5 μM; MG132). Loading control: HSP90. H-I. MYC and p-ERK1/2 protein expression of HCC44 cell line at different time points of indicated treatment (loading control: HSP90). Numbers correspond to relative MYC densitometry quantification. J. Relative MYC staining in tumors from T1-derived xenografts after 7-day treatment with indicated drugs (Soto: 30 mg/Kg; Mido: 25 mg/kg). Data: mean +/- SD. Test: one-way ANOVA, Dunnet's adjustment. K. Immunohistochemistry images of tumors in (J) stained for MYC. Scale bar: 200 uM.

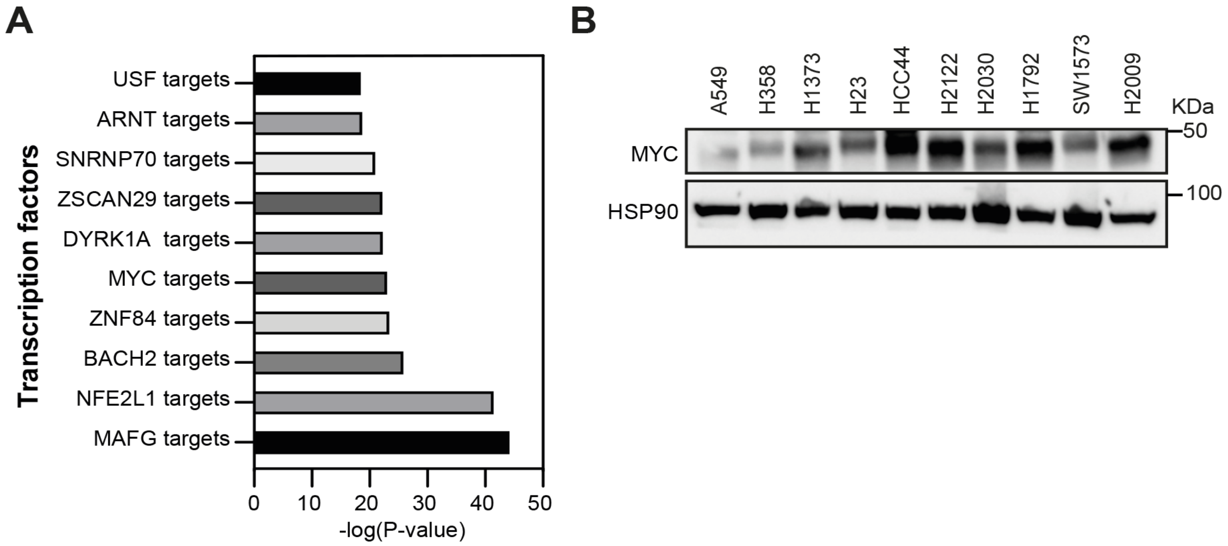

Suppl. Fig. 7. MYC upregulation renders mut *KRAS* cells resistant to Trametinib and Sotorasib. A. Potential transcriptional regulators of the upregulated Tram-resistant gene signature, obtained by MSigDB analysis. B. MYC protein expression in the indicated *KRAS* mut LUAD cells. HSP90 expression is shown as loading control.

References

1. Wilkerson MD et al. Differential pathogenesis of lung adenocarcinoma subtypes involving sequence mutations, copy number, chromosomal instability, and methylation. PLoS One 2012;7(5).

2. Okayama H et al. Identification of genes upregulated in ALK-positive and EGFR/KRAS/ALK-negative lung adenocarcinomas. Cancer Res. 2012;72(1):100–111.

3. Collisson EA et al. Comprehensive molecular profiling of lung adenocarcinoma: The cancer genome atlas research network. Nature 2014;511(7511):543–550.

4. Ding L et al. Somatic mutations affect key pathways in lung adenocarcinoma. Nature 2008;455(7216):1069–1075.

5. Beer DG et al. Gene-expression profiles predict survival of patients with lung adenocarcinoma. Nat. Med. 2002;8(8):816–824.

6. Chitale D et al. An integrated genomic analysis of lung cancer reveals loss of DUSP4 in EGFR-mutant tumors. Oncogene 2009;28(31):2773–2783.

UNCROPPED WESTERN BLOTS (Suppl. Figures)

Fig Suppl 3F

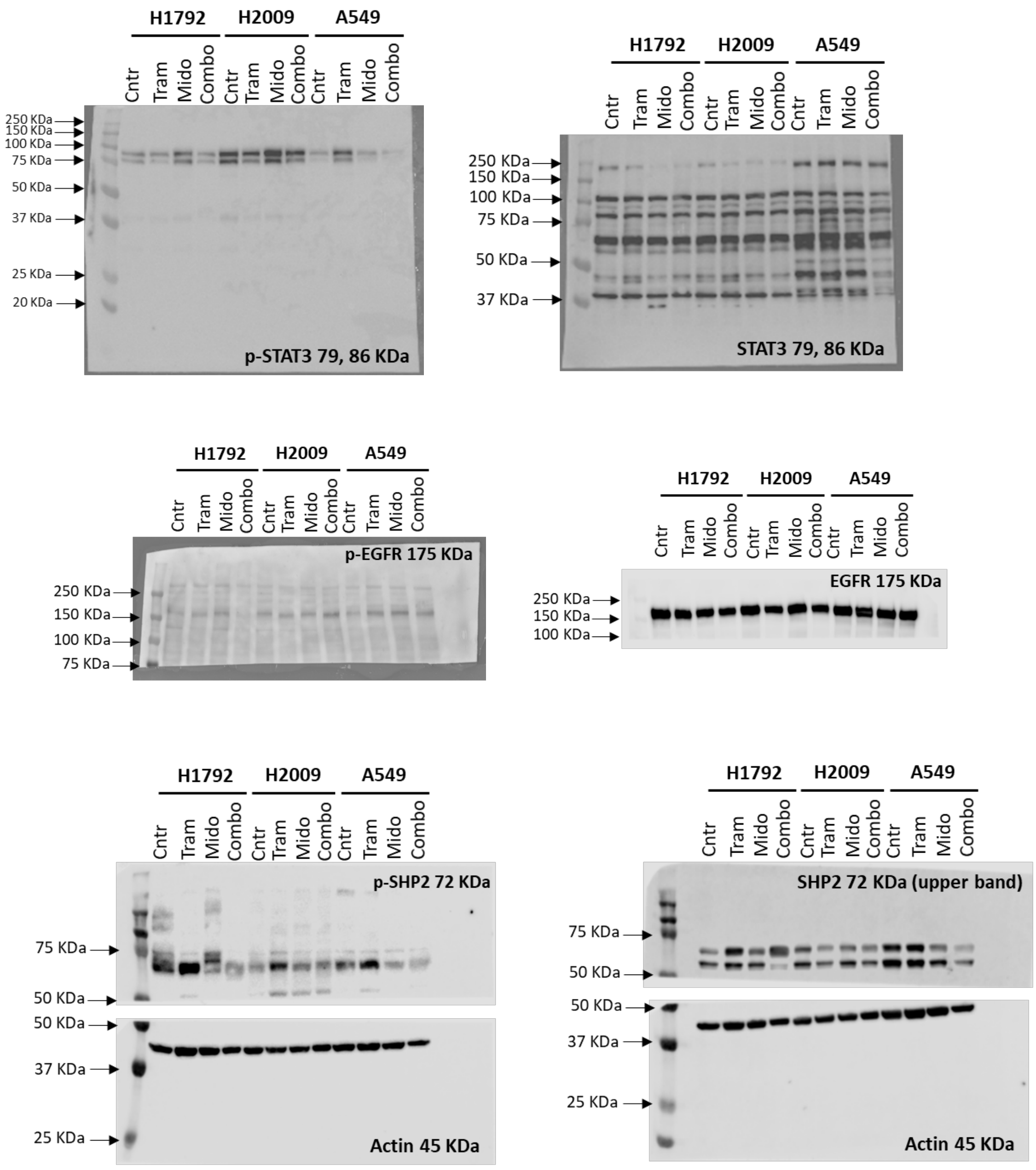

Fig Suppl 3K

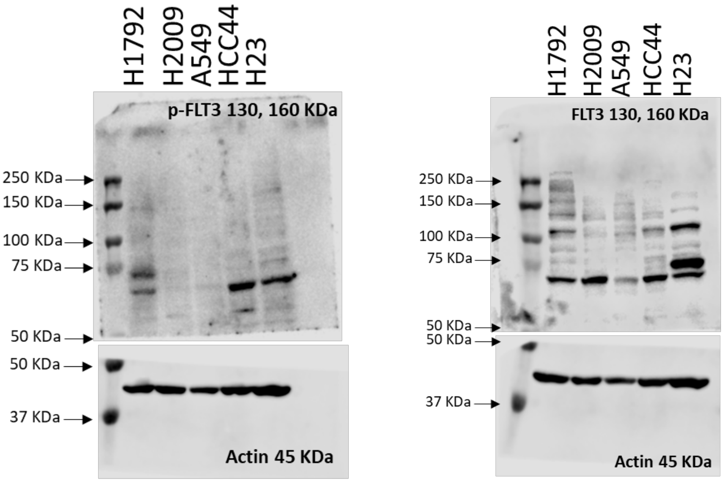

Fig Suppl 6G

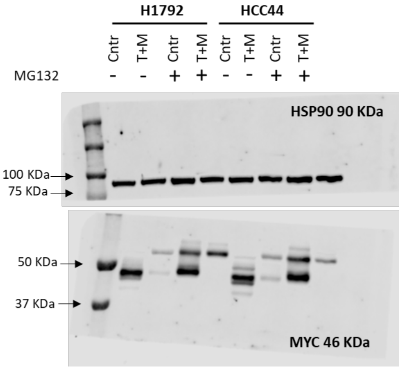

Fig Suppl 6H

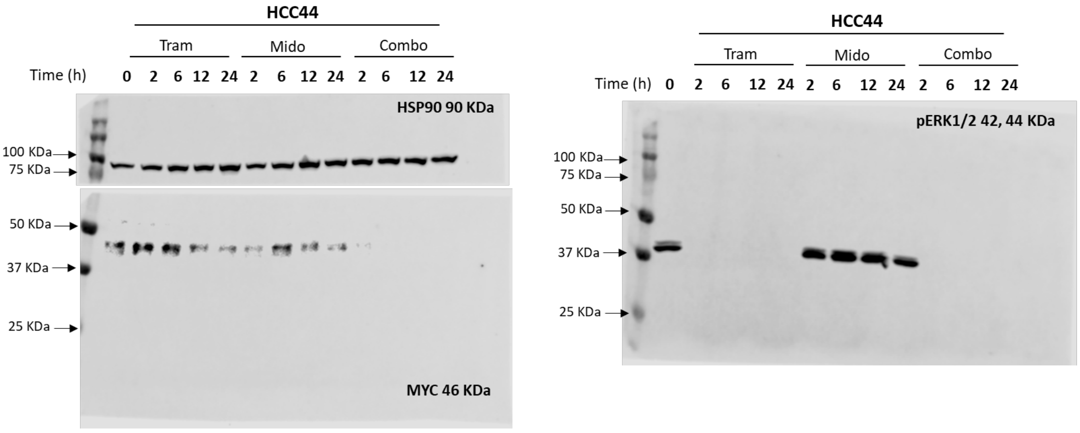

Fig Suppl 6I

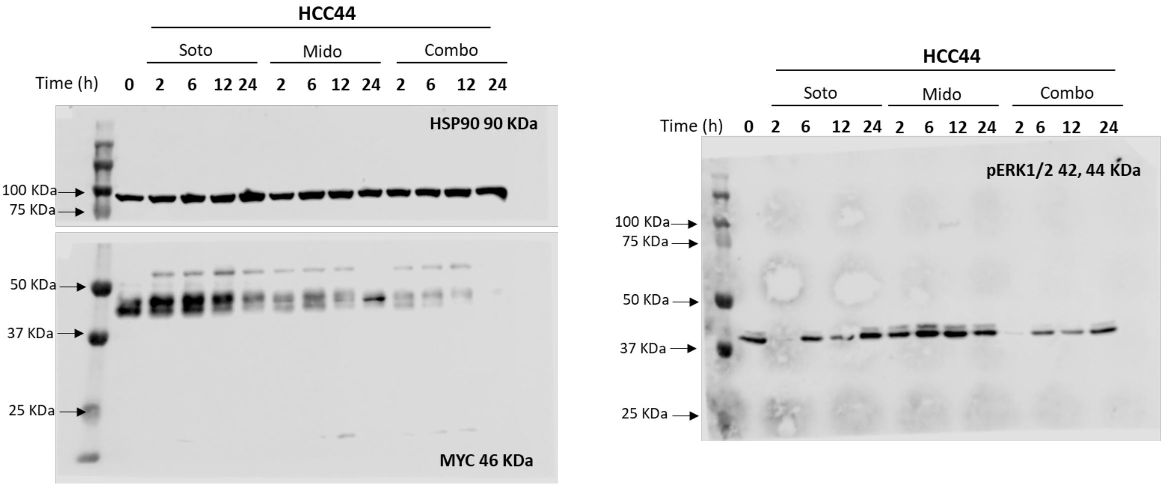

Fig Suppl 7B

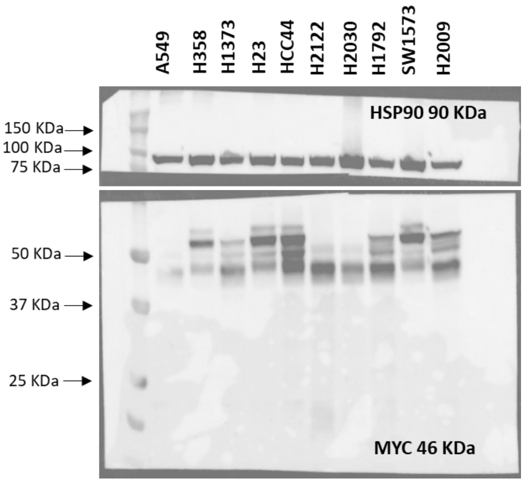

Supplement: Supplementary file 1 — Supplementary Information [file 41467_2023_41828_MOESM1_ESM.pdf]
