# Supplementary material for: Signature-driven repurposing of Midostaurin for combination with MEK1/2 and KRASG12C inhibitors in lung cancer
Source: Nat Commun. 2023 Oct 10;14:6332. doi: 10.1038/s41467-023-41828-z (PMC10564741; doi:10.1038/s41467-023-41828-z)
Supplement: Supplementary file 2 — Reporting Summary [file 41467_2023_41828_MOESM2_ESM.pdf]

nature portfolio

Corresponding author(s): Silvestre Vicent

Last updated by author(s): 21/08/23

Reporting Summary

Nature Portfolio wishes to improve the reproducibility of the work that we publish. This form provides structure for consistency and transparency in reporting. For further information on Nature Portfolio policies, see our Editorial Policies and the Editorial Policy Checklist.

Statistics

For all statistical analyses, confirm that the following items are present in the figure legend, table legend, main text, or Methods section.

n/a	Confirmed	
☐	☒	The exact sample size (*n*) for each experimental group/condition, given as a discrete number and unit of measurement
☐	☒	A statement on whether measurements were taken from distinct samples or whether the same sample was measured repeatedly
☐	☒	The statistical test(s) used AND whether they are one- or two-sided<br>*Only common tests should be described solely by name; describe more complex techniques in the Methods section.*
☒	☐	A description of all covariates tested
☐	☒	A description of any assumptions or corrections, such as tests of normality and adjustment for multiple comparisons
☐	☒	A full description of the statistical parameters including central tendency (e.g. means) or other basic estimates (e.g. regression coefficient) AND variation (e.g. standard deviation) or associated estimates of uncertainty (e.g. confidence intervals)
☐	☒	For null hypothesis testing, the test statistic (e.g. *F*, *t*, *r*) with confidence intervals, effect sizes, degrees of freedom and *P* value noted<br>*Give P values as exact values whenever suitable.*
☒	☐	For Bayesian analysis, information on the choice of priors and Markov chain Monte Carlo settings
☒	☐	For hierarchical and complex designs, identification of the appropriate level for tests and full reporting of outcomes
☒	☐	Estimates of effect sizes (e.g. Cohen's *d*, Pearson's *r*), indicating how they were calculated

Our web collection on statistics for biologists contains articles on many of the points above.

Software and code

Policy information about availability of computer code

Data collection	Connectivity Map<br>MaxQuant software<br>Image Studio Lite v5.2<br>SPECTROstar Nano v2.12<br>EPSON Scan v 3.9.3.4 ES<br>Leica Application Suite X (LAS X)<br>L1000 dataset in Library of Integrated Network-based Cellular Signatures (LINCS) (http://c3.lincscloud.org)<br>Uniprot proteome reference for Homo Sapiens (Proteome ID: UP000005640_9606, February 2019)<br>The studies for obtaining the interspecies KRAS signature are uploaded in GEO as GSE15325, GSE17671 and GSE49200
Data analysis	CompuSyn (www.combosyn.com)<br>GraphPad Prism 8<br>R statistical program (http:/www.R-project.org)<br>GSEA software<br>FloJo Software v9.3<br>Perseus software version 1.5.6.0<br>Metascape<br>3D Slicer Viewer Software

QuantStudio 3 Real-Time PCR

For manuscripts utilizing custom algorithms or software that are central to the research but not yet described in published literature, software must be made available to editors and reviewers. We strongly encourage code deposition in a community repository (e.g. GitHub). See the Nature Portfolio guidelines for submitting code & software for further information.

Data

Policy information about availability of data

All manuscripts must include a data availability statement. This statement should provide the following information, where applicable:

- Accession codes, unique identifiers, or web links for publicly available datasets
- A description of any restrictions on data availability
- For clinical datasets or third party data, please ensure that the statement adheres to our policy

DATA AVAILABILITY STATEMENT
The datasets generated during and/or analysed during the current study are available in public repositories. RNAseq files can be found at GSE161218 [https://www.ncbi.nlm.nih.gov/geo/query/acc.cgi?acc=GSE161218]. Search results files and MS raw data of proteomics analyses were deposited in the ProteomeXchange Consortium (http://proteomecentral.proteomexchange.org) via the PRIDE partner repository with the dataset identifiers PXD024023 [Project Webpage: http://www.ebi.ac.uk/pride/archive/projects/PXD024023; FTP Download: ftp://ftp.pride.ebi.ac.uk/pride/data/archive/2023/08/PXD024023]. Publicly available data sets used for this study are referenced in the text and figure legends (26-32). Source data are provided with this paper. The remaining data are available within the Article, Supplementary Information or Source Data file. Biological material (e.g. cell lines) generated in this study is available on the basis of a Material Transfer Agreement. Additional reagents will be made available upon reasonable request.

Human research participants

Policy information about studies involving human research participants and Sex and Gender in Research.

Reporting on sex and gender	The human data associated to the PDX used in this study are anonymized.
Population characteristics	*Describe the covariate-relevant population characteristics of the human research participants (e.g. age, genotypic information, past and current diagnosis and treatment categories). If you filled out the behavioural & social sciences study design questions and have nothing to add here, write "See above."*
Recruitment	*Describe how participants were recruited. Outline any potential self-selection bias or other biases that may be present and how these are likely to impact results.*
Ethics oversight	*Identify the organization(s) that approved the study protocol.*

Note that full information on the approval of the study protocol must also be provided in the manuscript.

Field-specific reporting

Please select the one below that is the best fit for your research. If you are not sure, read the appropriate sections before making your selection.

[x] Life sciences [ ] Behavioural & social sciences [ ] Ecological, evolutionary & environmental sciences

For a reference copy of the document with all sections, see nature.com/documents/nr-reporting-summary-flat.pdf

Life sciences study design

All studies must disclose on these points even when the disclosure is negative.

Sample size	Sample size was chosen using GPower (v3.1) or based on similar experiments previously published by the authors (Vallejo, Perurena and Guruceaga et al, Nat Comms, 2017; Valencia et al, JCI, 2020; Vallejo, Erice and Entrialgo-Cadierno et al, JHEP, 2021). For sample size estimation, the magnitude of effect was set up to 0.8, signification level was 0.05 and statistical power was 90%. Sex was considered as a variable for the estimation of the number of mice for in vivo experiments.
Data exclusions	No data were excluded from the analysis.
Replication	For in vitro experiments, at least 3 independent experiments were carried out with 2-6 replicates per experiment. All attempts at replication were successful. For in vivo experiments, the number of tumors per group was determined according the ethical guidelines for protocol number 057-18. As a general rule based on the triple R concept, in vivo experiments were not repeated. However, we did carry out some short-term in vivo experiments where the same drug response trend was observed.

Randomization	For experiments with mice, mice were randomized by their tumor size in order to have the same average tumor volume in all the groups at treatment start.
Blinding	Investigators were blinded to group allocation during in vivo data collection. Statistical analysis was done by an additional investigator who was also blinded to the groups.<br>No blinding was followed for in vitro experiments as the same person carried out the experiment and perfomed the subsequent analysis.

Reporting for specific materials, systems and methods

We require information from authors about some types of materials, experimental systems and methods used in many studies. Here, indicate whether each material, system or method listed is relevant to your study. If you are not sure if a list item applies to your research, read the appropriate section before selecting a response.

Materials & experimental systems

n/a	Involved in the study	
☐	☒	Antibodies
☐	☒	Eukaryotic cell lines
☒	☐	Palaeontology and archaeology
☐	☒	Animals and other organisms
☒	☐	Clinical data
☒	☐	Dual use research of concern

Methods

n/a	Involved in the study	
☒	☐	ChIP-seq
☐	☒	Flow cytometry
☒	☐	MRI-based neuroimaging

Antibodies

Antibodies used	β-TUBULIN: Santa Cruz Biotechnology, sc-9104<br>GAPDH: Abcam, ab9484<br>ACTIN: Sigma, A5441, lot# 079M4799V<br>HSP90: Santa Cruz Biotechnology, sc-69703, lot# D1316<br>KRAS: Sigma, WH0003845M1, lot# 17121-S2<br>MYC: Cell Signalling Technology, #5605, D84C12, Lot# 16<br>ERK1/2: Cell Signalling Technology, #9102, Lot# 28<br>p-ERK1/2: Cell Signalling Technology, #9101, Lot# 30<br>PARP: Cell Signalling Technology, #9542, Lot# 15<br>AKT: Cell Signalling Technology, #9272, Lot# 28<br>p-AKT: Cell Signalling Technology, #9271, Lot# 14<br>p70S6K: Cell Signalling Technology, #2708, Lot# 7<br>p-p70S6K: Cell Signalling Technology, #9205, Lot# 12<br>EGFR: Cell Signalling Technology, #2232, Lot# 15<br>p-EGFR: Cell Signalling Technology, #2236, Lot# 17<br>STAT3: Cell Signalling Technology, #4904, Lot# 13<br>p-STAT3: Cell Signalling Technology, #9145, Lot# 15<br>cJUN: Cell Signalling Technology, #9165, Lot# 13<br>SHP2: Cell Signalling Technology, #3397, Lot# 4<br>phospho-SHP2: Cell Signalling Technology, #3751, Lot# 4<br>FLT3: Cell Signalling Technology, #3461, Lot# 17<br>phospho-FLT3: Cell Signaling Technology, #3462, Lot# 19
Validation	β-TUBULIN: https://www.scbt.com/es/p/beta-tubulin-antibody-h-235<br>GAPDH: file:///C:/Users/imacayae/Downloads/datasheet_9484.pdf<br>ACTIN: https://www.thermofisher.com/order/genome-database/dataSheetPdf?producttype=antibody&productsubtype=antibody_primary&productId=MA5-15739&version=225<br>HSP90: https://datasheets.scbt.com/sc-69703.pdf<br>KRAS: https://www.sigmaaldrich.com/ES/es/product/sigma/wh0003845m1<br>MYC: https://www.cellsignal.com/datasheet.jsp?productId=5605&images=1&size=A4<br>ERK1/2: https://www.cellsignal.com/datasheet.jsp?productId=9102&images=1&size=A4<br>p-ERK1/2: https://www.cellsignal.com/datasheet.jsp?productId=9101&images=1&size=A4<br>PARP: https://www.cellsignal.com/datasheet.jsp?productId=9542&images=1&size=A4<br>AKT: https://www.cellsignal.com/datasheet.jsp?productId=9272&images=1&size=A4<br>p-AKT: https://www.cellsignal.com/datasheet.jsp?productId=9271&images=1&size=A4<br>p70S6K: https://www.cellsignal.com/datasheet.jsp?productId=2708&images=1&size=A4<br>p-p70S6K: https://www.cellsignal.com/datasheet.jsp?productId=9205&images=1&size=A4<br>EGFR: https://www.cellsignal.com/datasheet.jsp?productId=2232&images=1&size=A4<br>p-EGFR: https://www.cellsignal.com/datasheet.jsp?productId=2236&images=1&size=A4<br>STAT3: https://www.cellsignal.com/datasheet.jsp?productId=4904&images=1&size=A4

p-STAT3: https://www.cellsignal.com/datasheet.jsp?productId=9145&images=1&size=A4
cJUN: https://www.cellsignal.com/datasheet.jsp?productId=9165&images=1&size=A4
SHP2: https://www.cellsignal.com/datasheet.jsp?productId=3397&images=1&size=A4
phospho-SHP2: https://www.cellsignal.com/datasheet.jsp?productId=3751&images=1&size=A4
FLT3: AML cell lines lysates
phospho-FLT3: AML cell lines lysates

Eukaryotic cell lines

Policy information about cell lines and Sex and Gender in Research

Cell line source(s)

H1437: ATCC, Human, Male, Lung adenocarcinoma
H1568: ATCC, Human, Female, Lung adenocarcinoma
H2126: ATCC, Human, Male, Lung adenocarcinoma
HCC78: ATCC, Human, Male, Lung adenocarcinoma
H1650: ATCC, Human, Male, Lung adenocarcinoma
H1993: ATCC, Human, Female, Lung adenocarcinoma
H1792: ATCC, Human, Male, Lung adenocarcinoma
H2009: ATCC, Human, Female, Lung adenocarcinoma
A549: ATCC, Human, Male, Lung carcinoma
H358: ATCC, Human, Male, Lung carcinoma
H23: ATCC, Human, Male, Lung adenocarcinoma
HCC44: ATCC, Human, Female, Lung adenocarcinoma
CP435: General Hospital of Valencia, Human
H1373: ATCC, Human, Male, Adenocarcinoma
H2122: ATCC, Human, Female, Lung adenocarcinoma
H2030: ATCC, Human, Male, Lung adenocarcinoma
SW1573: ATCC, Human, Female, Lung carcinoma
H1792 Trametinib resistant: Generated in this study
H2009 Trametinib resistant: Generated in this study
A549 Trametinib resistant: Generated in this study
H23 Sotorasib resistant: Generated in this study
H358 Sotorasib resistant: Generated in this study
H2009-LacZ: Generated in this study
H2009-MYC overexpressed: Generated in this study
H23-LacZ: Generated in this study
H23-MYC overexpressed: Generated in this study
H358-LacZ: Generated in this study
H358-MYC overexpressed: Generated in this study
TP60 PDX: H12O, Human
TP79 PDX: H12O, Human
TP80 PDX: H12O, Human
TP181 PDX: H12O, Human
TP126 PDX: H12O, Human
KLA: CIMA, Mouse, Male, Lung cancer (https://doi.org/10.1172/JCI129012)
KLAp53ko: Generated in this study
T1: CNIO, Mouse, Female, Lung cancer, Generated in this study
T2: CNIO, Mouse, Female, Lung cancer, Generated in this study
T3: CNIO, Mouse, Female, Lung cancer, Generated in this study
220-1: CNIO, Mouse, Female, Lung cancer
220-2: CNIO, Mouse, Female, Lung cancer
95: CNIO, Mouse, Male, Lung cancer

Authentication

Human cancer cell lines were authenticated by the Genomics Unit at CIMA using Short Tandem Repeat profiling (AmpFLSTR® Identifiler® Plus PCR Amplification Kit). Mouse cell lines were authenticated by specific genomic PCRs.

Mycoplasma contamination

Cell lines were tested for mycoplasma contamination using the MycoAlert Mycoplasma Detection Kit (LONZA). Only mycoplasma-free cells were used.

Commonly misidentified lines
(See ICLAC register)

No commonly misidentifed cell lines were used in this study.

Animals and other research organisms

Policy information about studies involving animals; ARRIVE guidelines recommended for reporting animal research, and Sex and Gender in Research

Laboratory animals

Mouse, immune deficient Rag2-/-IL2R-/-, 8-12 week-old, female

	Mouse, KRasFSFG12C;Trp53FRT/FRT (mixed background, C57Bl/6J x 129/S4), 8-12 week-old<br><br>The University of Navarra- CIMA is an authorized User Center of experimental animals, registration number ES312010000132. Animal housing and experimentation is performed following existing regulation: Royal Decree 1201/2005 regarding the protection of animals used for experimental and other scientific purposes. The animal facilities are already prepared to house animals according to the European Directive 2010/63/EU. CIMA animal room facility (ARF) is maintained as a Specific Pathogen (SPF) facility. Good quality air at the appropriate temperature (20-24 degree C), humidity (50% +/-10%) and pressure levels are provided and monitored daily. A 12:12 light/dark cycle with progressive increase or decrease of light intensity to mimic the dawn/twilight is used across the facility.
Wild animals	The study did not involve wild animals.
Reporting on sex	Experiments with immundeficient mice were done with either male or female mice and sex was a variable considered for mice number estimation.
Field-collected samples	The study did not involve samples collected from the field.
Ethics oversight	Our research complies with all relevant ethics regulations and the project was approved by the Research Ethics Committee (CEI) of the University of Navarra under the protocol number 2020.010. Also, all experiments in mice were performed following ARRIVE guidelines and approved by the institutional Committee on Animal Research and Ethics of CIMA and CNIO under the protocol numbers 057-18 and PROEX 316/19.

Note that full information on the approval of the study protocol must also be provided in the manuscript.

Flow Cytometry

Plots

Confirm that:

☒ The axis labels state the marker and fluorochrome used (e.g. CD4-FITC).

☒ The axis scales are clearly visible. Include numbers along axes only for bottom left plot of group (a 'group' is an analysis of identical markers).

☒ All plots are contour plots with outliers or pseudocolor plots.

☒ A numerical value for number of cells or percentage (with statistics) is provided.

Methodology

Sample preparation	Cells were seeded and treated for 24 h with specified drugs. Then, cells were harvested and resuspended at 10^6 cells/mL in Annexin-binding buffer (10 mM HEPES, 140 mM NaCl and 2.5 mM CaCl2, pH 7.4). Cells were incubated with 3 µL of Alexa Fluor 647-conjugated Annexin-V and 15 µL of 7AAD for 15 minutes in the dark at RT. Finally, 400 µL of binding buffer were added to each tube and cells were acquired in FACSCanto II Cytometer (BD Biosciences).
Instrument	FACSCanto II Cytometer (BD Biosciences)
Software	FlowJo® software v9.3
Cell population abundance	Ten thousand cells of P1 population (On FSC/SSC plot) were aquired per sample.
Gating strategy	Alive and single cells were selected through FSC/SSC plots. Only 7AAD stained or only Alexa Fluor 647-Annexin V stained cells were used to select the positive populations. Results of experimental samples are ploted as 7ADD (Y axis) vs Annexin V (X axis).

☒ Tick this box to confirm that a figure exemplifying the gating strategy is provided in the Supplementary Information.
