# Supplementary material for: Signature-driven repurposing of Midostaurin for combination with MEK1/2 and KRASG12C inhibitors in lung cancer
Source: Nat Commun. 2023 Oct 10;14:6332. doi: 10.1038/s41467-023-41828-z (PMC10564741; doi:10.1038/s41467-023-41828-z)
Supplement: Supplementary file 5 — Description of Additional Supplementary Files [file 41467_2023_41828_MOESM5_ESM.pdf]

Title: Supplementary Data 1:

Description: Normalized RNAseq data_GSE161218

H1792 cell line treated with Trametinib and Lestaurtinib

Title: Supplementary data 2:

Description: Proteomics data_H1792 cell line_All comparisons

H1792 treated with Trametinib, Lestaurtinib or both

H1792-TR cell lines undergoing Trametinib treatment treated with Lestaurtinib
